# Brain‐derived neurotrophic factor acts at neurons of the subfornical organ to influence cardiovascular function

**DOI:** 10.14814/phy2.13704

**Published:** 2018-05-20

**Authors:** Emily A. E. Black, Pauline M. Smith, William McIsaac, Alastair V. Ferguson

**Affiliations:** ^1^ Department of Biomedical and Molecular Sciences Queen's University Kingston Ontario Canada; ^2^ Centre for Neuroscience Studies Queen's University Kingston Ontario Canada

**Keywords:** blood pressure, patch clamp, subfornical organ

## Abstract

Brain‐derived neurotrophic factor (BDNF), a neurotrophin traditionally associated with neural plasticity, has more recently been implicated in fluid balance and cardiovascular regulation. It is abundantly expressed in both the central nervous system (CNS) and peripheral tissue, and is also found in circulation. Studies suggest that circulating BDNF may influence the CNS through actions at the subfornical organ (SFO), a circumventricular organ (CVO) characterized by the lack of a normal blood–brain barrier (BBB). The SFO, well‐known for its involvement in cardiovascular regulation, has been shown to express BDNF mRNA and mRNA for the TrkB receptor at which BDNF preferentially binds. This study was undertaken to determine if: (1) BDNF influences the excitability of SFO neurons in vitro; and (2) the cardiovascular consequences of direct administration of BDNF into the SFO of anesthetized rats. Electrophysiological studies revealed that bath application of BDNF (1 nmol/L) influenced the excitability of the majority of neurons (60%, *n *=* *13/22), the majority of which exhibited a membrane depolarization (13.8 ± 2.5 mV,* n *=* *9) with the remaining affected cells exhibiting hyperpolarizations (−11.1 ± 2.3 mV,* n *=* *4). BDNF microinjections into the SFO of anesthetized rats caused a significant decrease in blood pressure (mean [area under the curve] AUC = −364.4 ± 89.0 mmHg × sec, *n *=* *5) with no effects on heart rate (mean AUC = −12.2 ± 3.4, *n *=* *5). Together these observations suggest the SFO to be a CNS site at which circulating BDNF could exert its effects on cardiovascular regulation.

## Introduction

Brain‐derived neurotrophic factor (BDNF), a member of the neurotrophin family, is abundantly expressed in both the central nervous system (CNS) and peripheral tissue, and circulates at concentrations correlated with levels found in the brain (Klein et al. 2011). Traditionally known for its actions in neural plasticity and processes related to learning and memory (Barde et al. 1982; Tyler et al. 2002) BNDF has, more recently, been suggested to play important roles in the regulation of other physiological processes including fluid balance (Arancibia et al. 2007) and cardiovascular regulation (Choe et al. 2015).

A role for BDNF actions at CNS structures to influence cardiovascular control is suggested by microinjection studies showing that direct administration of BDNF into the rostral ventrolateral medulla and medial nucleus tractus solitarius (mNTS), hindbrain regions involved in baroreflex control, or into the hypothalamic paraventricular nucleus (PVN) increases blood pressure (BP) and heart rate (HR; Wang and Zhou 2002; Clark et al. 2011; Erdos et al. 2015; Schaich et al. 2016). BDNF has been shown to directly act at the mNTS and PVN, as electrophysiological studies have shown that excitability of neurons from both structures is influenced by BDNF (Clark et al. 2011; McIsaac and Ferguson 2017).

BDNF has also been implicated in the central control of fluid balance, as secretion from the supraoptic nucleus (SON) has been shown to be increased following osmotic stress (Arancibia et al. 2007). Furthermore, BDNF mRNA expression in the subfornical organ (SFO), a CNS structure with well‐documented roles in fluid regulation (Buggy and Fisher 1974; Smith et al. 1995) is significantly increased when rats were either water deprived or chronically salt loaded (Saito et al. 2003; Hindmarch et al. 2008), suggesting that the SFO may be an important autonomic control center at which BDNF acts to exert its physiological effects.

The SFO is a CNS sensory circumventricular organ (CVO) characterized by the lack of a normal blood–brain barrier (BBB) and an abundance of peptidergic receptors, features that facilitate the detection of circulating signals from the periphery (Broadwell and Sofroniew 1993). The SFO is best known for its well‐established roles in the central control of fluid balance and cardiovascular regulation (Ishibashi and Nicolaidis 1981; Ferguson and Bains 1996; McKinley et al. 1998). Through its efferent projections to areas securely protected by the BBB, such as the SON, PVN, and the median preoptic area (MnPO), information conveyed to the SFO by large, lipophobic peripheral signals can be transmitted to these central autonomic control structures (Miselis 1981; Lind and Johnson 1982; Lind et al. 1982; Tanaka et al. 1985; Gutman et al. 1986).

Microarray analysis of SFO transcriptome revealed the presence of both BDNF mRNA and tyrosine receptor kinase B (TrkB) mRNA (Hindmarch et al. 2008), to which BDNF preferentially binds with high affinity (Pelleymounter et al. 1995). Interestingly, this transcriptome analysis revealed that fluid deprivation results in a more than fivefold increase in BDNF gene expression in the SFO (Hindmarch et al. 2008), the greatest change in any single gene's expression induced by dehydration in this structure. Together, these findings suggest that the SFO may be an important site for BDNF actions in the CNS.

Thus, this study was undertaken to determine the effect of BDNF on the excitability of SFO neurons and to investigate the effect of BDNF microinjection on cardiovascular control in anesthetized rats.

## Methods

### Animals

Male Sprague–Dawley rats, obtained from Charles River (PQ), were provided free access to food and water and maintained on a 12/12 light/dark cycle. All animal procedures were approved by the Queen's University Animal Care Committee and are in accordance with the guidelines of the Canadian Council on Animal Care.

### Cell culture preparation

Dissociated SFO neurons were prepared from 21 to 25 days male SD rats as previously described (Smith et al. 2009). Rats were first anaesthetized by inhalation of isoflurane in a bell chamber and then decapitated using a guillotine. The brain was removed from the skull and immediately placed in ice‐cold, carbogenated, artificial cerebral spinal fluid (aCSF) which measured 280–300 mOsm, had a pH of 7.2 (adjusted using NaOH) and contained (in mmol/L): NaCl (124), KCl (2.5), KH_2_PO4 (1.24), CaCl_2_ (2.27), MgSO_4_ (1.3), NaHCO_3_ (19) and glucose (10). A tissue chunk containing SFO and the surrounding hippocampal commissure was quickly dissected and the SFO was microdissected away from all surrounding tissue and placed in a Hibernate‐A solution (Gibco, Gaithersburg, MD) that has been supplemented with B27 (Invitrogen, Carlsbad, CA). The isolated SFO tissue from three rats was then combined and placed in 5 mL of hibernate media (Brain Bits, Springfield, IL) containing 2 mg/mL papain (Worthington Biochemical, Lakewood, NJ) and placed in a water bath for 30 min at 31°C. The papain solution was removed, the tissue washed, and then triturated with hibernate in order to gently dissociate neurons from surrounding connective tissue. Once the cells had been collected, they were spun down in a centrifuge at 200*g* at 4°C for 8 min, the supernatant removed, and the pellet of cells gently resuspended using Neurobasal‐A (Gibco) supplemented with B27 containing 100 U/mL penicillin‐streptomycin (Invitrogen) and 0.5 mmol/L l‐glutamine (Invitrogen). The cells were then plated (12 *μ*L) onto 35 mm plastic bottom dishes (MatTek, Ashland, MA) and placed in the CO_2_ incubator for 2.5 h at 37°C at which time 1.5 mL of Neurobasal‐A was added to each dish. Cells were maintained in the incubator for 1–4 days prior to electrophysiological recordings.

### SFO slice preparation

In order to confirm that the observed effects of BDNF on membrane excitability in dissociated SFO neurons was not a consequence of the dissociation procedure or the length of time in synaptic isolation, BDNF was bath applied to SFO neurons in acute slice preparations. Rats (21–35 days) were quickly decapitated using a guillotine and the brain was removed and placed into ice‐old oxygenated (95% O_2_–5% CO_2_) slicing solution which consists of (in mmol/L): NaCl (87), KCl (2.5), NaHCO_3_ (25), CaCl_2_ (0.5), MgCl_2_ (7), NaH_2_PO_4_ (1.25), glucose (25), and sucrose (75). A block of tissue containing the SFO was isolated, and 300 *μ*m coronal slices were obtained using a vibratome (Leica, Nussloch Germany). Slices were then incubated at 31°C for at least 1 h in oxygenated aCSF composed of (in mmol/L):NaCl (126), KCl (2.5), NaHCO_3_ (26), CaCl_2_ (2), MgCl_2_ (2), NaH_2_PO_4_ (1.25),glucose (10), and a pH of 7.2 (adjusted using NaOH) prior to electrophysiological recording.

### Electrophysiological techniques

Whole‐cell patch‐clamp recordings of SFO neurons were acquired using a Multiclamp 700B patch‐clamp amplifier (Molecular Devices, San Jose, CA). The stimulation and recording parameters were controlled using SPIKE2, version 7.05b, and SIGNAL, version 4.08 (Cambridge Electronics Design, Cambridge, UK). Whole‐cell recordings in current clamp configuration were filtered at 2.4 kHz using a Cambridge Electronics Design Micro 1401 interface 9 (Cambridge Electronics Design) and sampled at 10 kHz. Electrodes were made of borosilicate glass with an inner diameter of 0.68 mm and outer diameter of 1.2 mm (World Precision Instruments, Sarasota, FL) pulled using a Flaming Brown micropipette puller (P47; Sutter Instrument Company, Novato, CA). Electrodes were then filled with internal recording solution, which measured 280–300 mOsm, had a pH of 7.2 (adjusted using KOH) and contained (in mmol/L): potassium gluconate (125), MgCl∙6H_2_O (2), ethylene glycol tetraacetic acid (1.0), KCl (10), NaATP (2), HEPES (10) and CaCl_2_ (0.3), with a free Ca^2+^ concentration of 65 nmol/L. Only electrodes having a resistance of 2–5 MΩ when filled with internal pipette solution were used for recording.

Dissociated SFO neurons in the 35 mm culture dishes in which they were maintained were placed on a stable recording platform. The cells were perfused with recording aCSF (37°C) at a rate of 1.5 mL/min. The aCSF measured 280–300 mOsm, had a pH of 7.2 (adjusted using NaOH) and contained (in mmol/L): NaCl (140), KCl (5), MgCl_2_ (1), CaCl_2_ (2), HEPES (10), mannitol (5), and glucose (5). SFO slices were relocated to a recording chamber perfused with carbogenated slice recording aCSF heated to 31°C at a flow rate of 1.5–2.5 mL/min. An upright differential interference contrast microscope at ×40 was used to visualize neurons (Scientifica). In both cases (dissociated cells and tissue slices), a micromanipulator (MP‐225; Sutter Instrument Company) was used to place the electrode in the bath and slowly lowered to touch the cell membrane of the selected neuron. Gentle negative pressure was applied in order to form a GΩ seal, and whole‐cell patch configuration was obtained by applying a brief and consistent pulse of negative pressure and, using current clamp configuration, the baseline membrane potential was recorded. Recordings from dissociated cells were also obtained using the perforated patch configuration. Amphotericin‐B (Sigma‐Aldrich, Oakville, ON, Canada) was used as the perforating agent and was dissolved in dimethyl sulfoxide (DMSO; Sigma‐Aldrich). Electrodes were first dipped in normal internal recording solution for ~1 sec, and then backfilled with the amphotericin‐containing (~536 *μ*g/mL) internal recording solution. As with the whole‐cell technique, the electrode was slowly lowered to make contact with the cell membrane and negative pressure was applied to form a GΩ seal. At this point, the amphotericin was given 10–30 min to perforate the cell membrane and allow access.

### Electrophysiology data analysis

In order for a cell to be included in our analysis it had to display action potentials (spontaneous or evoked) of greater than 60 mV and a minimum 300 sec stable baseline membrane potential prior to BDNF application. In addition, for effects to be classified as responses they either showed a return toward baseline or a minimum 5 min stable membrane potential at the peak of the observed effect. The calculated liquid junction potential was determined to be 15 mV and was subtracted from all recordings. All values are reported as mean ± the standard error of the mean (SEM).

### Assessment of BNDF effects

Once a stable baseline membrane potential was established, BDNF (50 pmol/L–2 nmol/L; Phoenix Pharmaceuticals) was bath applied for 60 sec followed by a return to aCSF. Mean membrane potential in 100s bins both before (baseline) and after peptide application was calculated. The effect(s) of BDNF application on the membrane potential of a given cell was determined by subtracting the control baseline membrane potential from the mean membrane potential during the 100s period immediately following peptide administration displaying the greatest change from the mean baseline membrane potential. In order for a response to be considered significant, the change in membrane potential had to be greater than twice the standard deviation (SD) of the baseline membrane potential measured over the 100 sec control period (total of 100 × 10 K = 1 million data points).

### In vivo microinjections

Urethane anesthetized (1.4 g/kg) male Sprague–Dawley rats (150–300 g) were fitted with femoral arterial catheters for the measurement of blood pressure and heart rate. Body temperature was maintained at 37°C throughout the duration of the experiment using a feedback controlled heating blanket. Animals were placed in a stereotaxic frame and a midline incision was made to expose the surface of the skull. A small burr hole was then drilled such that a microinjection cannula (150 *μ*m tip diameter; Rhodes Medical Instruments, Summerland, CA) could be advanced into the region of SFO according to the coordinates of Paxinos and Watson (Paxinos and Watson 1982). Following a minimum 2 min stable baseline recording period, BDNF (2 or 20 nmol/L) was delivered as a bolus (0.5 *μ*L) into the region by a pressure driven 5 *μ*L Hamilton microsyringe over 10 sec and the effects on BP and HR assessed.

At the conclusion of the experiment, animals were overdosed with anesthetic and perfused with 0.9% saline, followed by 10% formalin, through the left ventricle of the heart. The brain was removed and placed in formalin for a minimum of 24 h after which, 100 *μ*m coronal sections were cut through the region of SFO using a vibratome. These sections were mounted, cresyl violet stained, and the anatomical location of the microinjection site was verified at the light microscope level by an observer unaware of the experimental protocol or the data obtained.

### In vivo data analysis

Animals were placed into one of two anatomical groups (SFO or non‐SFO) according to the histological location of the microinjection site. SFO sites were those in which the tip of the injection pipette was located within the anatomical boundaries of SFO, whereas non‐SFO sites included animals where this tip was located at least 0.2 mm from the border of this structure. Normalized BP and HR data (mean baseline BP and HR was calculated for 60 sec prior to injection and subtracted from all data points prior to and post injection) were obtained for each animal 60 sec prior to the time of microinjection (control period) until 120 sec post microinjection. The area under the curve (AUC, area between baseline and each BP or HR response) was then calculated for each animal for the 120 sec time period immediately following the injection and the mean AUC was then calculated for both the SFO and non‐SFO group. In order to determine whether cardiovascular responses elicited by BDNF were dose dependent, a higher concentration (20 nmol/L) of BDNF was microinjected into the SFO of separate groups of animals. A one‐way anova (followed by a Tukey's Multiple Comparison Test post hoc analysis) was used to determine if cardiovascular changes elicited by BDNF microinjection were significant depending upon anatomical location of the microinjection site (SFO or non‐SFO) or concentration of BDNF (2 or 20 nmol/L) delivered. [Correction added on 22 May 2018, after first online publication: Missing numbers indicating molarity of solutions in the Methods section have now been included.]

## Results

### BDNF influences excitability of dissociated SFO neurons

**Figure 1:**
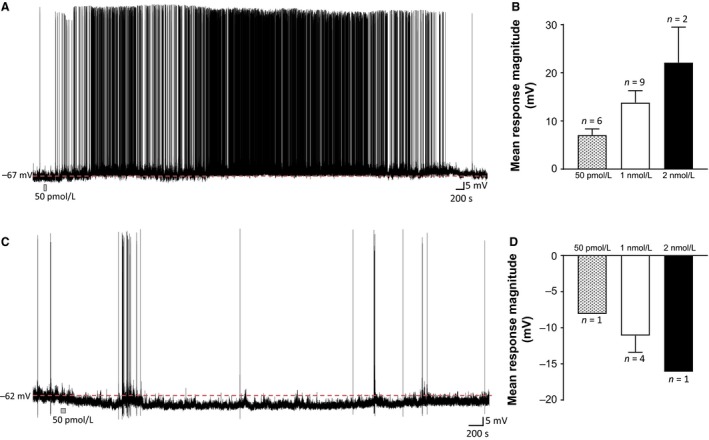
BDNF affects SFO neuronal excitability. Current clamp recordings from two different dissociated SFO neurons illustrating a depolarization (A) and a hyperpolarization (C) in response to 50 pmol/L BDNF. Time and duration of BDNF application is indicated by the gray bar at the bottom of each recording. The bar graphs to the right of each trace show the mean change in membrane potential observed in response to increasing concentrations of BDNF for cells that depolarized (C) and hyperpolarized (D). Errors bars represent SEM. BDNF, brain‐derived neurotrophic factor; SFO, subfornical organ.

Initial experiments were performed at a concentration of 2 nmol/L, as this concentration had been previously shown to influence the excitability of CNS neurons (McIsaac and Ferguson 2017). Bath application at this concentration influenced the excitability in the majority (3/4) of SFO neurons tested, eliciting large depolarizations in two of the affected cells (22.4 ± 7.4 mV, *n *=* *2; see Fig. 1), effects that stabilized at this depolarized membrane potential but did not recover during the length of our recordings. The remaining affected cell displayed a large hyperpolarization (−16.1 mV). Due to the magnitude and duration of these responses, the concentration of BDNF was decreased to 1 nmol/L, which was also found to influence the majority (60%) of neurons tested (*n *=* *13/22). Most of the neurons influenced by BDNF (9/13) exhibited a depolarizing response (13.8 ± 2.5 mV, *n *=* *9; see Fig. 1), whereas a smaller proportion of affected neurons responded with a hyperpolarization (−11.1 ± 2.3 mV, *n *=* *4; see Fig. 1). While the majority of recordings at 1 nmol/L were performed using the whole‐cell technique (*n *=* *20), recordings were also obtained using perforated patch and similar responses observed, although much longer recording times were possible using this technique, and thus all additional tests were carried out using this technique.

An additional 19 cells were tested at 50 pmol/L BDNF and 37% of neurons tested (*n *=* *7/19) showed a significant change in membrane potential, with depolarizations observed in six cells (7.1 ± 1.3 mV, *n *=* *6), and the remaining cell exhibiting a hyperpolarization (−8.1 mV *n *=* *1) as shown in Figure 1, a similar distribution of response types to the higher (1 nmol/L) BDNF concentration (*P *=* *0.29, Chi‐square). These responses were typically of shorter duration with a complete return to baseline membrane potential, when observed (5/7), occurring 3–180 min after return to aCSF.

### BDNF influences excitability of SFO neurons in slice preparations

In order to confirm that the influence of BDNF on the excitability of SFO neurons is intrinsic to the cells, and not a result of the dissociation process or the length of time the cells were in synaptic isolation, we performed patch‐clamp recordings on four SFO neurons in slice preparation at 2 nmol/L BDNF. As in the dissociated preparation, membrane depolarizations (12.6 ± 0.7 mV, *n *=* *2) and hyperpolarizations (−25.9 mV, *n *=* *1) were observed in response to BDNF, while one cell was unaffected.

### Microinjection of BDNF into the SFO has hypotensive effects

**Figure 2:**
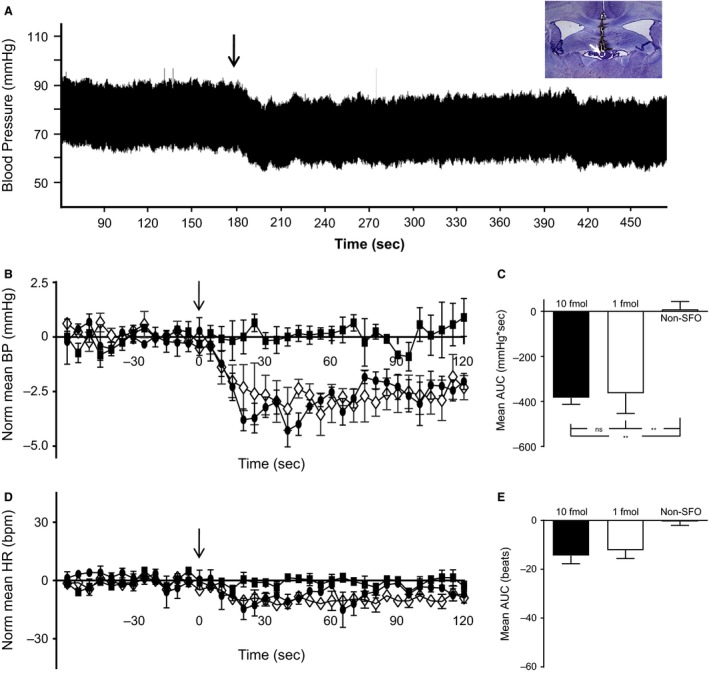
BDNF Microinjection into the SFO elicits decreases in blood pressure. (A) Raw BP recording from a single animal showing the BP response elicited by 10 fmol (0.5 *μ*L of 20 nmol/L) BDNF. Time of injection is indicated by the arrow. The inset photomicrograph shows the microinjection location within the SFO. (B–E) Normalized mean BP (B) and HR (D) from animals with BDNF microinjection sites in SFO (closed circle 10fmol; open diamond 1 fmol) and non‐SFO (closed square). Time of injection is indicated by the arrows. Bar graphs to the right show mean AUC for BP (C) and HR (D) responses to microinjection of 10fmol (*n *=* *5, solid bar) or 1 fmol (*n *=* *6, open bar) BDNF into SFO and non‐SFO (*n *=* *3 hatched bar) locations. BP: anova *P *=* *0.0032 Tukey post hoc analysis: **P <* *0.01. BDNF, brain‐derived neurotrophic factor; SFO, subfornical organ; AUC, area under the curve.

A total of 21 animals were used, 11 of which were shown to have microinjection sites histologically localized to SFO (see Fig. 2), whereas three microinjection locations were outside the anatomical boundaries of SFO (non‐SFO). The remaining seven animals were excluded from analysis as the microinjection locations could not be reliably classified as SFO or non‐SFO sites.

Microinjection of 10 fmol (0.5 *μ*L of 20 nmol/L) BDNF into the SFO resulted in rapid decreases in mean BP (mean AUC = −364.4 ± 89.0 mmHg × sec, *n *=* *5), without effect on HR (mean AUC = −12.2 ± 3.4 beats, *n *=* *5; see Fig. 2). These hypotensive effects usually returned to baseline within 300 sec of BDNF application. As illustrated in Figure 2, these depressor effects were not found to be dose dependent as 1 fmol (0.5 *μ*L of 2 nmol/L) BDNF microinjection into the SFO elicited similar BP and HR responses (BP: mean AUC = −384.3 ± 28.5 mmHg × sec, *n *=* *6; HR: mean AUC = −14.4 ± 3.3 beats, *n *=* *6; see Fig. 2) to the larger BDNF concentration. Cardiovascular responses elicited as a consequence of BDNF administration were, however, site specific as BDNF microinjection into non‐SFO sites were without effect on BP (mean AUC = 9.4 ± 34.4 mmHg × sec, *n *=* *3) or HR (mean AUC = −0.46 ± 1.52 beats, *n *=* *3). Baseline BP and HR was not different between the groups (BP: 2 nmol/L SFO: 84.9 ± 5.7 mmHg, *n *=* *6; 20 nmol/L SFO: 94.1 ± 5.3 mmHg, *n *=* *5; non‐SFO: 80.2 ± 2.4 mmHg, *n *=* *3, anova *P *=* *0.27: HR: 2 nmol/L SFO: 477.2 ± 18.7 BPM, *n *=* *6; 20 nmol/L SFO: 474.2 ± 10.4 BPM, *n *=* *5; non‐SFO: 434.8 ± 8.8 BPM, *n *=* *3, anova *P *=* *0.23).

## Discussion

In this study, we investigated the cellular effects of BDNF on the excitability of SFO neurons as well as the cardiovascular consequences of direct administration of this peptide into the SFO of anesthetized rats.

Our findings that SFO neurons respond to BDNF are the first to demonstrate electrophysiological consequences of BDNF actions in the SFO. Bath administration of BDNF influenced the excitability of the majority of SFO neurons in both our dissociated cells and in our slice preparation. The effects of BDNF on our dissociated neurons indicate that BDNF directly influences SFO neurons, as this preparation leaves neurons devoid of neuronal input. We can be confident that the observed neuronal responses are not the result of the dissociation process or the length of time in isolation, as similar responses were observed in acute slice preparations in which neuronal connections had been maintained.

These electrophysiological studies demonstrated that BDNF influenced the membrane potential of SFO neurons in either an excitatory or inhibitory fashion, whereas a proportion of neurons did not respond to BDNF. This heterogeneous response profile is not uncommon in the SFO (Smith et al. 2009; Kuksis and Ferguson 2014; Cancelliere and Ferguson 2017). These different response types may represent different anatomical groups of neurons within the SFO such as interneurons or projection neurons. For example, a subset of BDNF responsive SFO neurons may correspond to those that project to areas that are known to be involved in fluid homeostasis, such as the MnPO, and raphe nuclei (Lind 1986; Oldfield et al. 1991), whereas a second population may represent those involved in cardiovascular regulation, such as the lateral hypothalamic area (Swanson and Lind 1986; Allen and Cechetto 1992) or suprachiasmatic nucleus (Lind et al. 1982; Krout et al. 2002; Scheer et al. 2003), and an additional subset to areas known to be involved in the regulation of both physiological processes, such as the PVN (Miselis 1981; Ferguson et al. 1984a,b; Li and Ferguson 1993). While this study clearly demonstrates electrophysiological actions of BDNF at the SFO, future studies using techniques such as single‐cell PCR or retrograde tracing to investigate the neuronal phenotypes associated with each of the response profiles observed would certainly be of interest to determine the potential physiological consequences of BDNF actions at these SFO neurons.

BDNF has been shown to exert its effects via actions at the TrkB receptor (Kang et al. 1996) and mRNA for this receptor is expressed in the SFO (Hindmarch et al. 2008). Future investigations utilizing a TrkB antagonist such as ANA‐12 (Cazorla et al. 2011) are necessary in order to establish whether the actions of BDNF at the SFO are a result of this neurotrophin interacting with this receptor.

A long‐lasting elevation in intracellular Ca^2+^ has been shown to occur when BDNF binds to TrkB in neurons of the hippocampus (Berninger et al. 1993), visual cortex (Mizoguchi et al. 2002) and in pontine neurons (Li et al. 1999), an effect likely resulting from a signal transduction pathway that activates phospholipase C. Thus, the long duration effects on membrane excitability observed in SFO neurons are not unexpected and may be the result of the mechanisms described above.

A functional role for BDNF in the SFO has been suggested in previous studies that demonstrate mRNA expression of this neurotrophin is increased in this CVO under conditions of osmotic stress such as salt loading and dehydration (Saito et al. 2003; Hindmarch et al. 2008). Our in vivo experiments demonstrated that direct administration of BDNF into the SFO decreased BP without affecting HR, a finding that suggests cardiovascular effects are not due to modulation of the baroreceptor reflex nor a removal of sympathetic tone. Although these responses were not shown to be concentration dependent at the doses used in this study, they were site specific as BDNF microinjection into areas outside the anatomical boundaries of SFO were without effect. We are confident that the effects on BP were a result of BDNF actions in the SFO, as we have previously demonstrated that vehicle injections into the SFO do not alter BP (Smith et al. 2007; Smith and Ferguson 2012; Dai et al. 2013).

The hypotensive effects observed following BDNF administration into SFO are in contrast to the effects of BDNF microinjection into the PVN, mNTS, and RVLM (Wang and Zhou 2002; Clark et al. 2011; Erdos et al. 2015; Schaich et al. 2016), areas protected by the BBB, and to that of icv administration (Wang et al. 2012). This finding is not unprecedented as a variety of centrally acting vasoactive peptides have been shown to have opposing effects when microinjected into the SFO compared to ventricular administration or microinjection into CNS sites protected by the BBB (Smith et al. 2007; Smith and Ferguson 2012; Dai et al. 2013). These dichotomous findings suggest that BDNF may have both hormonal and neurotransmitter roles. The depressor effects elicited by BDNF actions at the SFO may represent a hormonal role for BDNF in central cardiovascular control as the fenestrated capillaries of the SFO allow circulating substances access to this CNS structure.

A role for BDNF in a variety of physiological processes outside that of its traditional role in neural plasticity have been proposed. BDNF has been suggested to play a role in energy homeostasis as BDNF heterozygous mutant mice are obese while icv injection of BDNF into the anteroventral third ventricle induces significant weight loss in these mutant mice (Kernie et al. 2000). These same mutant mice demonstrate an insensitivity to both insulin and leptin (Kernie et al. 2000), peptides that have been shown to influence the activity of SFO neurons (Smith et al. 2009; Lakhi et al. 2013). A functional role for BDNF in the SFO has been suggested by the findings that demonstrated the expression of BDNF mRNA is drastically altered by either dehydration, food deprivation, or osmotic stress (Saito et al. 2003; Hindmarch et al. 2008) suggesting the SFO to be an important structure for mediating the responses to a variety of physiological challenges.

Circulating BDNF concentrations correlate with hippocampal concentrations (Klein et al. 2011), and exercise appears to reverse the decreased BDNF mRNA expression observed in hippocampal tissue in response to stress (Zheng et al. 2006). Interestingly, peripheral levels of BDNF in humans are shown to increase during bouts of prolonged exercise (Rasmussen et al. 2009; Zoladz and Pilc 2010). Given its involvement in exercise, our observation that BDNF acts at the SFO to decrease BP suggests that perhaps BDNF may act as a regulatory molecule to attenuate increased BP during periods of sustained physical activity.

In summation, the results of this study support the conclusion that neurons exist within the SFO that are able to detect circulating BDNF and that the SFO may be a site at which circulating BDNF acts to influence cardiovascular control, likely in part through BP regulation. Interestingly, in addition to cardiovascular regulation and fluid balance, both the SFO and BDNF have been shown to be involved in the regulation of other physiological processes such as immune function (Takahashi et al. 1997; Desson and Ferguson 2003; Trott and Harrison 2014; Xiao et al. 2016) and metabolic regulation (Pelleymounter et al. 1995; Smith et al. 2009), suggesting that the SFO may represent a CNS site at which BDNF acts in an integrative manner to control these autonomic systems.

## References

[phy213704-bib-0001] Allen, G. V. , and D. F. Cechetto . 1992 Functional and anatomical organization of cardiovascular pressor and depressor sites in the lateral hypothalamic area: I. Descending projections. J. Comp. Neurol. 315:313–332. 1740546 10.1002/cne.903150307

[phy213704-bib-0002] Arancibia, S. , A. Lecomte , M. Silhol , E. Aliaga , and L. Tapia‐Arancibia . 2007 In vivo brain‐derived neurotrophic factor release and tyrosine kinase B receptor expression in the supraoptic nucleus after osmotic stress stimulus in rats. Neuroscience. 146:864–873. 17346893 10.1016/j.neuroscience.2007.01.057

[phy213704-bib-0003] Barde, Y. A. , D. Edgar , and H. Thoenen . 1982 Purification of a new neurotrophic factor from mammalian brain. EMBO J. 1:549–553. 7188352 10.1002/j.1460-2075.1982.tb01207.x PMC553086

[phy213704-bib-0004] Berninger, B. , D. E. Garcia , N. Inagaki , C. Hahnel , and D. Lindholm . 1993 BDNF and NT‐3 induce intracellular Ca^2+^ elevation in hippocampal neurones. NeuroReport. 4:1303–1306. 7505114 10.1097/00001756-199309150-00004

[phy213704-bib-0005] Broadwell, R. D. , and M. V. Sofroniew . 1993 Serum proteins bypass the blood‐brain fluid barriers for extracellular entry to the central nervous system. Exp. Neurol. 120:245–263. 8491281 10.1006/exnr.1993.1059

[phy213704-bib-0006] Buggy, J. , and A. E. Fisher . 1974 Evidence for a dual center role for angiotensin in water and sodium intake. Nature. 250:733–735. 4370006 10.1038/250733a0

[phy213704-bib-0007] Cancelliere, N. M. , and A. V. Ferguson . 2017 Subfornical organ neurons integrate cardiovascular and metabolic signals. Am. J. Physiol. Regul. Integr. Comp. Physiol. 312:R253–R262. 28003212 10.1152/ajpregu.00423.2016

[phy213704-bib-0008] Cazorla, M. , J. Premont , A. Mann , N. Girard , C. Kellendonk , and D. Rognan . 2011 Identification of a low‐molecular weight TrkB antagonist with anxiolytic and antidepressant activity in mice. J. Clin. Invest. 121:1846–1857. 21505263 10.1172/JCI43992 PMC3083767

[phy213704-bib-0009] Choe, K. Y. , S. Y. Han , P. Gaub , B. Shell , D. L. Voisin , B. A. Knapp , et al. 2015 High salt intake increases blood pressure via BDNF‐mediated downregulation of KCC2 and impaired baroreflex inhibition of vasopressin neurons. Neuron. 85:549–560. 25619659 10.1016/j.neuron.2014.12.048 PMC4577058

[phy213704-bib-0010] Clark, C. G. , E. M. Hasser , D. L. Kunze , D. M. Katz , and D. D. Kline . 2011 Endogenous brain‐derived neurotrophic factor in the nucleus tractus solitarius tonically regulates synaptic and autonomic function. J. Neurosci. 31:12318–12329. 21865474 10.1523/JNEUROSCI.0746-11.2011 PMC3408222

[phy213704-bib-0011] Dai, L. , P. M. Smith , M. Kuksis , and A. V. Ferguson . 2013 Apelin acts in the subfornical organ to influence neuronal excitability and cardiovascular function. J. Physiol. 591:3421–3432. 23629509 10.1113/jphysiol.2013.254144 PMC3717236

[phy213704-bib-0012] Desson, S. E. , and A. V. Ferguson . 2003 Interleukin 1beta modulates rat subfornical organ neurons as a result of activation of a non‐selective cationic conductance. J. Physiol. 550:113–122. 12879863 10.1113/jphysiol.2003.041210 PMC2343005

[phy213704-bib-0013] Erdos, B. , I. Backes , M. L. McCowan , L. F. Hayward , and D. A. Scheuer . 2015 Brain‐derived neurotrophic factor modulates angiotensin signaling in the hypothalamus to increase blood pressure in rats. Am. J. Physiol. Heart Circ. Physiol. 308:H612–H622. 25576628 10.1152/ajpheart.00776.2014 PMC4360054

[phy213704-bib-0014] Ferguson, A. V. , and J. S. Bains . 1996 Electrophysiology of the circumventricular organs. Front. Neuroendocrinol. 17:440–475. 8905349 10.1006/frne.1996.0012

[phy213704-bib-0015] Ferguson, A. V. , T. A. Day , and L. P. Renaud . 1984a Subfornical organ stimulation excites paraventricular neurons projecting to dorsal medulla. Am. J. Physiol. 247:R1088–R1092. 6095687 10.1152/ajpregu.1984.247.6.R1088

[phy213704-bib-0016] Ferguson, A. V. , T. A. Day , and L. P. Renaud . 1984b Subfornical organ efferents influence the excitability of neurohypophysial and tuberoinfundibular paraventricular nucleus neurons in the rat. Neuroendocrinology. 39:423–428. 6096750 10.1159/000124015

[phy213704-bib-0017] Gutman, M. B. , J. Ciriello , and G. J. Mogenson . 1986 Electrophysiological identification of forebrain connections of the subfornical organ. Brain Res. 382:119–128. 3533207 10.1016/0006-8993(86)90118-6

[phy213704-bib-0018] Hindmarch, C. , M. Fry , S. T. Yao , P. M. Smith , D. Murphy , and A. V. Ferguson . 2008 Microarray analysis of the transcriptome of the subfornical organ in the rat: regulation by fluid and food deprivation. Am. J. Physiol. Regul. Integr. Comp. Physiol. 295:R1914–R1920. 18832082 10.1152/ajpregu.90560.2008

[phy213704-bib-0019] Ishibashi, S. , and S. Nicolaidis . 1981 Hypertension induced by electrical stimulation of the subfornical organ (SFO). Brain Res. Bull. 6:135–139. 7470958 10.1016/s0361-9230(81)80038-x

[phy213704-bib-0020] Kang, H. , L. Z. Jia , K. Y. Suh , L. Tang , and E. M. Schuman . 1996 Determinants of BDNF‐induced hippocampal synaptic plasticity: role of the Trk B receptor and the kinetics of neurotrophin delivery. Learn. Mem. 3:188–196. 10456089 10.1101/lm.3.2-3.188

[phy213704-bib-0021] Kernie, S. G. , D. J. Liebl , and L. F. Parada . 2000 BDNF regulates eating behavior and locomotor activity in mice. EMBO J. 19:1290–1300. 10716929 10.1093/emboj/19.6.1290 PMC305670

[phy213704-bib-0022] Klein, A. B. , R. Williamson , M. A. Santini , C. Clemmensen , A. Ettrup , M. Rios , et al. 2011 Blood BDNF concentrations reflect brain‐tissue BDNF levels across species. Int. J. Neuropsychopharmacol. 14:347–353. 20604989 10.1017/S1461145710000738

[phy213704-bib-0023] Krout, K. E. , J. Kawano , T. C. Mettenleiter , and A. D. Loewy . 2002 CNS inputs to the suprachiasmatic nucleus of the rat. Neuroscience. 110:73–92. 11882374 10.1016/s0306-4522(01)00551-6

[phy213704-bib-0024] Kuksis, M. , and A. V. Ferguson . 2014 Cellular actions of nesfatin‐1 in the subfornical organ. J. Neuroendocrinol. 26:237–246. 24612143 10.1111/jne.12143

[phy213704-bib-0025] Lakhi, S. , W. Snow , and M. Fry . 2013 Insulin modulates the electrical activity of subfornical organ neurons. NeuroReport. 24:329–334. 23481267 10.1097/WNR.0b013e32835ffc14

[phy213704-bib-0026] Li, Z. , and A. V. Ferguson . 1993 Subfornical organ efferents to paraventricular nucleus utilize angiotensin as a neurotransmitter. Am. J. Physiol. 265:R302–R309. 8103642 10.1152/ajpregu.1993.265.2.R302

[phy213704-bib-0027] Li, H. S. , X. Z. Xu , and C. Montell . 1999 Activation of a TRPC3‐dependent cation current through the neurotrophin BDNF. Neuron. 24:261–273. 10677043 10.1016/s0896-6273(00)80838-7

[phy213704-bib-0028] Lind, R. W. 1986 Bi‐directional, chemically specified neural connections between the subfornical organ and the midbrain raphe system. Brain Res. 384:250–261. 3779379 10.1016/0006-8993(86)91161-3

[phy213704-bib-0029] Lind, R. W. , and A. K. Johnson . 1982 Subfornical organ‐median preoptic connections and drinking and pressor responses to angiotensin II. J. Neurosci. 2:1043–1051. 7108583 10.1523/JNEUROSCI.02-08-01043.1982 PMC6564271

[phy213704-bib-0030] Lind, R. W. , G. W. Van Hoesen , and A. K. Johnson . 1982 An HRP study of the connections of the subfornical organ of the rat. J. Comp. Neurol. 210:265–277. 7142442 10.1002/cne.902100306

[phy213704-bib-0031] McIsaac, W. , and A. V. Ferguson . 2017 Glucose concentrations modulate brain‐derived neurotrophic factor responsiveness of neurones in the paraventricular nucleus of the hypothalamus. J. Neuroendocrinol. 29:1–10. 10.1111/jne.12464 28258626

[phy213704-bib-0032] McKinley, M. J. , A. M. Allen , P. Burns , L. M. Colvill , and B. J. Oldfield . 1998 Interaction of circulating hormones with the brain: the roles of the subfornical organ and the organum vasculosum of the lamina terminalis. Clin. Exp. Pharmacol. Physiol. Suppl. 25:S61–S67. 9809195 10.1111/j.1440-1681.1998.tb02303.x

[phy213704-bib-0033] Miselis, R. R. 1981 The efferent projections of the subfornical organ of the rat: a circumventricular organ within a neural network subserving water balance. Brain Res. 230:1–23. 7317773 10.1016/0006-8993(81)90388-7

[phy213704-bib-0034] Mizoguchi, Y. , A. Monji , and J. Nabekura . 2002 Brain‐derived neurotrophic factor induces long‐lasting Ca2 + ‐activated K+ currents in rat visual cortex neurons. Eur. J. Neurosci. 16:1417–1424. 12405954 10.1046/j.1460-9568.2002.02198.x

[phy213704-bib-0035] Oldfield, B. J. , R. R. Miselis , and M. J. McKinley . 1991 Median preoptic nucleus projections to vasopressin‐containing neurones of the supraoptic nucleus in sheep. A light and electron microscopic study. Brain Res. 542:193–200. 1851451 10.1016/0006-8993(91)91566-j

[phy213704-bib-0036] Paxinos, G. , and C. Watson . 1982 The rat brain in stereotaxic coordinates. Academic Press, New York. 10.1016/0165-0270(80)90021-7 6110810

[phy213704-bib-0037] Pelleymounter, M. A. , M. J. Cullen , and C. L. Wellman . 1995 Characteristics of BDNF‐induced weight loss. Exp. Neurol. 131:229–238. 7534721 10.1016/0014-4886(95)90045-4

[phy213704-bib-0038] Rasmussen, P. , P. Brassard , H. Adser , M. V. Pedersen , L. Leick , E. Hart , et al. 2009 Evidence for a release of brain‐derived neurotrophic factor from the brain during exercise. Exp. Physiol. 94:1062–1069. 19666694 10.1113/expphysiol.2009.048512

[phy213704-bib-0039] Saito, J. , Y. Ozaki , H. Ohnishi , T. Nakamura , and Y. Ueta . 2003 Osmotic stimuli increase brain‐derived neurotrophic factor mRNA level in the rat subfornical organ. Neurosci. Lett. 347:65–68. 12873729 10.1016/s0304-3940(03)00614-1

[phy213704-bib-0040] Schaich, C. L. , T. L. Wellman , B. Koi , and B. Erdos . 2016 BDNF acting in the hypothalamus induces acute pressor responses under permissive control of angiotensin II. Auton. Neurosci. 197:1–8. 26948539 10.1016/j.autneu.2016.02.011 PMC9387676

[phy213704-bib-0041] Scheer, F. A. , A. Kalsbeek , and R. M. Buijs . 2003 Cardiovascular control by the suprachiasmatic nucleus: neural and neuroendocrine mechanisms in human and rat. Biol. Chem. 384:697–709. 12817466 10.1515/BC.2003.078

[phy213704-bib-0042] Smith, P. M. , and A. V. Ferguson . 2012 Cardiovascular actions of leptin in the subfornical organ are abolished by diet‐induced obesity. J. Neuroendocrinol. 24:504–510. 22103447 10.1111/j.1365-2826.2011.02257.x

[phy213704-bib-0043] Smith, P. M. , R. J. Beninger , and A. V. Ferguson . 1995 Subfornical organ stimulation elicits drinking. Brain Res. Bull. 38:209–213. 7496814 10.1016/0361-9230(95)00088-v

[phy213704-bib-0044] Smith, P. M. , W. K. Samson , and A. V. Ferguson . 2007 Cardiovascular actions of orexin‐A in the rat subfornical organ. J. Neuroendocrinol. 19:7–13. 17184481 10.1111/j.1365-2826.2006.01497.x

[phy213704-bib-0045] Smith, P. M. , A. P. Chambers , C. J. Price , W. Ho , C. Hopf , K. A. Sharkey , et al. 2009 The subfornical organ: a central nervous system site for actions of circulating leptin. Am. J. Physiol. Regul. Integr. Comp. Physiol. 296:R512–R520. 19020290 10.1152/ajpregu.90858.2008

[phy213704-bib-0046] Swanson, L. W. , and R. W. Lind . 1986 Neural projections subserving the initiation of a specific motivated behavior in the rat: new projections from the subfornical organ. Brain Res. 379:399–403. 3742231 10.1016/0006-8993(86)90799-7

[phy213704-bib-0047] Takahashi, Y. , P. Smith , A. Ferguson , and Q. J. Pittman . 1997 Circumventricular organs and fever. Am. J. Physiol. 273:R1690–R1695. 9374811 10.1152/ajpregu.1997.273.5.R1690

[phy213704-bib-0048] Tanaka, J. , H. Kaba , H. Saito , and K. Seto . 1985 Electrophysiological evidence that circulating angiotensin II sensitive neurons in the subfornical organ alter the activity of hypothalamic paraventricular neurohypophyseal neurons in the rat. Brain Res. 342:361–365. 4041838 10.1016/0006-8993(85)91137-0

[phy213704-bib-0049] Trott, D. W. , and D. G. Harrison . 2014 The immune system in hypertension. Adv. Physiol. Educ. 38:20–24. 24585465 10.1152/advan.00063.2013 PMC4459918

[phy213704-bib-0050] Tyler, W. J. , M. Alonso , C. R. Bramham , and L. D. Pozzo‐Miller . 2002 From acquisition to consolidation: on the role of brain‐derived neurotrophic factor signaling in hippocampal‐dependent learning. Learn. Mem. 9:224–237. 12359832 10.1101/lm.51202 PMC2806479

[phy213704-bib-0051] Wang, H. , and X. F. Zhou . 2002 Injection of brain‐derived neurotrophic factor in the rostral ventrolateral medulla increases arterial blood pressure in anaesthetized rats. Neuroscience. 112:967–975. 12088754 10.1016/s0306-4522(02)00085-4

[phy213704-bib-0052] Wang, M. F. , Y. C. Chan , H. T. Lee , and L. Z. Hong . 2012 Regulation of the intracerebroventricular administration of brain‐derived neurotrophic factor on baroreflex function and insulin sensitivity in rats. Chin. J. Physiol. 55:184–191. 22784283 10.4077/CJP.2012.BAA015

[phy213704-bib-0053] Xiao, R. , S. M. Bergin , W. Huang , A. M. Slater , X. Liu , R. T. Judd , et al. 2016 Environmental and Genetic Activation of Hypothalamic BDNF Modulates T‐cell Immunity to Exert an Anticancer Phenotype. Cancer Immunol. Res. 4:488–497. 27045020 10.1158/2326-6066.CIR-15-0297 PMC4891265

[phy213704-bib-0054] Zheng, H. , Y. Liu , W. Li , B. Yang , D. Chen , X. Wang , et al. 2006 Beneficial effects of exercise and its molecular mechanisms on depression in rats. Behav. Brain Res. 168:47–55. 16290283 10.1016/j.bbr.2005.10.007 PMC2662337

[phy213704-bib-0055] Zoladz, J. A. , and A. Pilc . 2010 The effect of physical activity on the brain derived neurotrophic factor: from animal to human studies. J. Physiol. Pharmacol. 61:533–541. 21081796

